# The International *Oryza* Map Alignment Project (I*O*MAP): the Americas—past achievements and future directions

**DOI:** 10.1093/jxb/erac490

**Published:** 2022-12-17

**Authors:** Aseel Alsantely, Rafal Gutaker, María E Navarrete Rodríguez, Griselda Arrieta-Espinoza, Eric J Fuchs, Antonio Costa de Oliveira, Joe Tohme, Andrea Zuccolo, Rod A Wing, Alice Fornasiero

**Affiliations:** Center for Desert Agriculture, Biological and Environmental Sciences & Engineering Division (BESE), King Abdullah University of Science and Technology (KAUST), Thuwal 23955-6900, Saudi Arabia; Royal Botanic Gardens, Kew, Kew Green, Richmond, Surrey TW9 3AE, UK; Center for Desert Agriculture, Biological and Environmental Sciences & Engineering Division (BESE), King Abdullah University of Science and Technology (KAUST), Thuwal 23955-6900, Saudi Arabia; Centro de Investigación en Biología Celular y Molecular, Universidad de Costa Rica, Ciudad de la Investigación-C.P., San José 11501-2050, Costa Rica; Escuela de Biología, Universidad de Costa Rica, San José 11501-2060, Costa Rica; Plant Genomics and Breeding Center, Eliseu Maciel School of Agronomy, Federal University of Pelotas, Pelotas-RS, Brazil; International Center for Tropical Agriculture (CIAT), Cali 763537, Colombia; Center for Desert Agriculture, Biological and Environmental Sciences & Engineering Division (BESE), King Abdullah University of Science and Technology (KAUST), Thuwal 23955-6900, Saudi Arabia; Crop Science Research Center, Sant’Anna School of Advanced Studies, Pisa 56127, Italy; Center for Desert Agriculture, Biological and Environmental Sciences & Engineering Division (BESE), King Abdullah University of Science and Technology (KAUST), Thuwal 23955-6900, Saudi Arabia; Arizona Genomics Institute, School of Plant Sciences, University of Arizona, Tucson, AZ 85721, USA; Center for Desert Agriculture, Biological and Environmental Sciences & Engineering Division (BESE), King Abdullah University of Science and Technology (KAUST), Thuwal 23955-6900, Saudi Arabia; University of Trento, Italy

**Keywords:** Biodiversity conservation, genetic diversity, herbaria specimens, *in situ* specimens, I*O*MAP, neodomestication, population genetics, resistance traits, wild relatives of rice in the Americas

## Abstract

The wild relatives of rice hold unexplored genetic diversity that can be employed to feed an estimated population of 10 billion by 2050. The *Oryza* Map Alignment Project (*O*MAP) initiated in 2003 has provided comprehensive genomic resources for comparative, evolutionary, and functional characterization of the wild relatives of rice, facilitating the cloning of >600 rice genes, including those for grain width (GW5) and submergence tolerance (SUB1A). Following in the footsteps of the original project, the goal of ‘I*O*MAP: the Americas’ is to investigate the present and historic genetic diversity of wild *Oryza* species endemic to the Americas through the sequencing of herbaria and *in situ* specimens. The generation of a large diversity panel describing past and current genetic status and potential erosion of genetic variation in the populations will provide useful knowledge for the conservation of the biodiversity in these species. The wild relatives of rice in the Americas present a wide range of resistance traits useful for crop improvement and neodomestication approaches. In the race against time for a sustainable food future, the neodomestication of the first cereal species recently accomplished in *O. alta* opens the door to the potential neodomestication of the other wild *Oryza* species in Americas.

## Teaser

We review the relevance and major outcomes of I*O*MAP to the wild *Oryza*species in the Americas, focusing on biodiversity conservation, and discuss achievements and future implications of the neodomestication of *Oryza alta*.

## Introduction

Rice is one of the major cereal grains and a staple food for more than half of the world’s population (Bhandari, 2019). Rice is a significant food source representing 19% of per capita calorie consumption across the globe (Maclean *et al.*, 2013), and its by-products (e.g. straw and husks) can be used to produce biofuels, paper, or fertilizers (Goodman, 2020). Asian rice (*Oryza sativa*) is the most consumed rice species, with China, India, Thailand, Vietnam, and Pakistan accounting for 74% of the global export (Bhandari, 2019). According to the United States Department of Agriculture (USDA), the world rice production in 2020/2021 was 509.3 Mt, with China and India generating 53.5% of the global production (https://apps.fas.usda.gov/). The domestication of Asian rice began ~9000 years ago in East Asia, possibly in the Yangtze valley in China, where *O. sativa* was domesticated from *O. rufipogon* (Molina *et al.*, 2011). In contrast, African rice (*O. glaberrima*) is a minor crop grown in limited areas of West Africa and Suriname (South America). The domestication of *O. glaberrima* started ~3000 years ago in Africa with the cultivation of *O. barthii* along the delta of the Niger River and its later development into the modern African rice (Wang *et al.*, 2014). In addition to the domesticated rice species, the *Oryza* genus contains 25 wild species distributed in the pan-tropics, which exhibit tremendous diversity in morphology, agronomic traits, and adaptations to different biotic and abiotic stress (Sanchez *et al.*, 2013). The genus spans 15 million years (MYs) of evolutionary history, and includes species with different ploidy levels and genome architectures. Morphological, cytological, and molecular analyses permitted classification of the *Oryza* species into 11 genome types, six diploid (i.e. AA, BB, CC, EE, FF, and GG, 2*n*=2*x*=24 chromosomes), and five tetraploid (i.e. BBCC, CCDD, HHJJ, HHKK, and KKLL, 2*n*=4*x*=48 chromosomes). Based on their phylogenetic relationships and crossability with cultivated rice, *Oryza* species are further classified into three gene pools (Harlan and de Wet, 1971; Vaughan, 1989). The primary gene pool consists of eight species belonging to the AA genome type; the two cultivated species and six wild species (i.e. *O. barthii*, *O. longistaminata, O. meridionalis*, *O. glumipatula*, *O. nivara*, and *O. rufipogon*; however, the latter three species are often considered as synonyms). The AA genome species are also regarded as the *O. sativa* complex, one of the four *Oryza* complexes defined by the taxonomic studies of Tateoka in the 1960s (Tateoka, 1963). Species belonging to the *O. sativa* complex are characterized by high crossability, with wild species commonly used for trait introgression into rice cultivars via traditional breeding methods. The rate of successful hybridization between cultivated rice and species belonging to the secondary and tertiary gene pools is progressively lower given higher reproductive barriers and cross incompatibilities. The secondary gene pool comprises *O. brachyantha* (FF) and the *O. officinalis* complex, which contains 11 wild species and five genome types, namely BB (*O. punctata*); CC (*O. eichingeri*, *O. officinalis*, and *O. rhizomatis*); EE (*O. australiensis*); BBCC (*O. malampuzhaensis*, *O. minuta*, and *O. schweinfurthiana*); and CCDD (*O. alta*, *O. grandiglumis*, and *O. latifolia*). The tertiary gene pool includes the *O. meyeriana* complex (comprising the GG type *O. meyeriana* and *O. granulata*), the *O. ridleyi* complex (comprising the HHJJ type *O. ridleyi* and *O. longiglumis*), and the unclassified species *O. schlecteri* (HHKK) and *O. coarctata* (KKLL) (Vaughan, 1989).

The wild relatives of rice harbor large (and often unexplored) genetic variation that can potentially be used for crop improvement, and ultimately food security (Dempewolf *et al.*, 2017; Brar and Khush, 2018). Through millions of years of evolution and genetic adaptation to different environments, the wild *Oryza* species have accumulated allelic diversity in genes useful for crop improvement, such as biotic stress resistance (e.g. insects, fungi, and bacteria), abiotic stress tolerance (e.g. wounding, salinity, cold, and flooding), and nutritional- and yield-related traits (Song *et al.*, 2005). However, the utility of wild relatives for rice improvement depends on the availability of these genetic resources: *in situ* and *ex situ* conservation strategies are therefore crucial to preserve the wild relatives from extinction and to counteract the anthropogenic activity on their natural habitats (Medeiros *et al.*, 2021).

The *Oryza* Map Alignment Project (*O*MAP) and the *Oryza* Genome Evolution Project (*O*GEP) were established almost two decades ago to provide comprehensive genomic resources for comparative, evolutionary, and functional characterization of the wild relatives of rice. This platform has allowed the discovery of novel genetic diversity in wild species to generate new rice genotypes with improved traits (Wing *et al.*, 2007). The project was further extended to several international research alliances under the frame of the International *Oryza* Map Alignment Project (I*O*MAP; Jacquemin *et al.*, 2013). The resources provided by I*O*MAP have allowed the generation of many advanced mapping populations through conventional trait introgression and molecular breeding techniques (Gutierrez *et al*., 2010; Ram *et al.*, 2010; Bocco *et al.*, 2012). With the development of genome editing and CRISPR/Cas [clustered regularly interspaced palindromic repeats(CRISPR)/CRISPR-associated protein] technology (Jinek *et al*., 2012, 2013; Doudna and Charpentier, 2014), an innovative approach called neodomestication (Gutaker *et al.*, 2022) arose as a promising alternative to conventional crop improvement (Nooryazdan *et al.*, 2011; Zsögön *et al.*, 2017; Li *et al.*, 2018). The recent publication of a high-quality genome reference of *O. alta* and a road-map for the rapid domestication of this species represents the first description of a neodomesticated polyploid cereal (Yu *et al.*, 2021).

Whereas several studies subsequent to the I*O*MAP gave insights into Asian and African *Oryza* species’ evolutionary history (Ammiraju *et al.*, 2007; Chen *et al.*, 2013; Stein *et al.*, 2018), a description of the genetic diversity of the wild *Oryza* species in the Americas is lagging behind. The project ‘I*O*MAP: the Americas’ was funded to investigate the genetic diversity of the wild relatives of rice endemic to the Americas. The project aims to provide a comprehensive description of the status of modern wild *Oryza* species by generating an extensive collection of resequenced specimens collected in North, Central, and South America. The project’s perspective is to inform on both the historical and current biodiversity status of the wild rice species endemic to the Americas, and identify putative candidates for neodomestication (Fornasiero *et al.*, 2022).

Here, we review the primary outcomes of I*O*MAP and present major methods and goals of the project ‘I*O*MAP: the Americas’, giving a background on the evolutionary origin of the wild *Oryza* in the Americas, their biotic and abiotic resistance traits, their conservation status, and the recent neodomestication of *O. alta*.

## Relevance and major outcomes of the I*O*MAP

The first digital platform of the *Oryza* genus generated in the 2000s by the *O*MAP and *O*GEP consortia (Wing *et al.*, 2007; Ammiraju *et al.*, 2010a) included the release of BAC-end sequence/fingerprint-based physical maps of 17 species spanning all eight AA-genome species and one of each of the other nine extant genome types (i.e. BB, CC, BBCC, CCDD, EE, FF, GG, KKLL, and HHJJ). Alignment of the genome sequences against the International Rice Genome Sequencing Project (IRGSP) reference genome sequence allowed the characterization of transposable element (TE) abundance, distribution, and evolutionary dynamics (Piegu *et al.*, 2006; Ammiraju *et al*., 2007, 2010b; Roulin *et al.*, 2008), the study of the evolution of gene families (Ammiraju *et al.*, 2008; Lu *et al.*, 2009), and the characterizations of mutations and genome-wide rearrangements (Hurwitz *et al.*, 2010). Ammiraju *et al.* (2008) sequenced a set of orthologous loci harboring the alcohol-dehydrogenase 1 and 2 genes (*Adh-1* and *Adh-2*, two established loci for comparative genomic studies across plant species) in the six extant diploid genome types, and compared them with the *Adh1* locus from the IRGSP reference sequence (Os*Adh1*RefSeq). Comparative analysis showed that the locus underwent recent rearrangements leading to rapid and lineage-specific diversification of the *Oryza* species analyzed, with gain and loss of gene family members and general synteny disruption. The analysis of TE insertion events at the Adh1 region revealed that long terminal repeat retrotransposable elements (LTR-RTs) are not conserved in the array of *Oryza* species and appeared to have inserted independently in the different genome types within the last 5 MYs after speciation (Ammiraju *et al.*, 2008).

The goal of I*O*MAP was to produce reference genome sequences and transcriptome data for the 17 original species and generate a genome-scale and genus-wide platform available to the rice scientific community to address major questions related to comparative genomics. The primary foci were to: (i) gain new knowledge at the macro-evolutionary level (i.e. understand the dynamics of the evolution of genomes and polyploidy in the genus *Oryza*, unravel the history and impact of rice domestication, and explore the diversity and evolution of gene families useful for rice improvement); (ii) develop advanced mapping populations for functional and breeding purposes; and (iii) carry out population genomics and ecological studies for biodiversity conservation purposes (Jacquemin *et al.*, 2013).

The vast array of genomic tools and data produced by I*O*MAP set the basis for generating chromosome-level reference assemblies of many *Oryza* species (Yu *et al.*, 2002; International Rice Genome Sequencing Project and Sasaki, 2005; Chen *et al.*, 2013; Zhang *et al.*, 2015a). The comparative analysis of 13 *Oryza* reference genomes, including the AA genome species, *O. punctata*, *O. brachyantha*, and *Leersia perrieri* used as the outgroup, revealed important features of the evolution of the *Oryza* genomes (Stein *et al.*, 2018). Using >6000 single-copy orthologs, Stein and colleagues inferred a phylogeny of 13 single-individual species in which the AA clade was estimated to diverge 2.5 MYs ago and the diversification occurred at a rate of ~0.50 net new species per MY. Analysis of TEs revealed lineage-specific amplification and rapid turnover of LTR-RTs in the genomes under study. Comparative annotation and phylogenetic analysis of LTR-RTs showed that the genomes underwent recent and lineage-specific bursts of transposition. By tracking 75 orthologous LTR-RT loci across the eight most closely related AA species, Stein *et al.* estimated an average loss rate of 3.62 kb per MY per LTR-RT. At the genome scale, this implies that a quarter of an AA genome would be eliminated within 3–4 MYs in the absence of new bursts of transposition. Comparative analysis of gene annotations showed that lineage-specific gene families in the Oryzeae clade exhibited features of newly emerging genes, such as shorter coding sequences, lower expression, more rapid evolution rates (i.e. relaxed purifying selection), and evolutionary instability compared with gene families emerged in the common ancestors of grasses (*Poaceae*). However, *Oryzeae*-specific loci showed a high degree of conserved synteny even among distantly related species in the clade (Stein *et al.*, 2018).

Finally, the generation and release of high-quality reference genomes of cultivated and non-cultivated rice species by I*O*MAP has allowed the cloning of >600 rice genes, many of which are not present in the IRGSP reference itself, including those for grain width (GW5), and submergence tolerance (SUB1A; Wing, 2015).

## I*O*MAP: the Americas

**Fig. 1.**
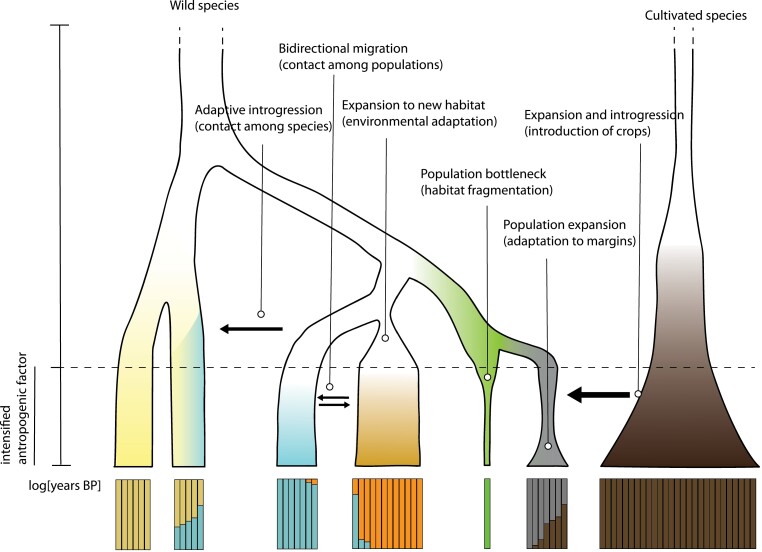
Anthropogenic activities, such as agriculture, and natural events affect the interaction between the wild species and its native environment, altering the connections with other species, and causing genetic threats (i.e. drift, inbreeding, and hybridization). Human intervention can have a high impact on the demography and structure of the populations and can lead to (from left to right): new contacts among species/populations; expansion to new habitats; habitat fragmentation; population expansion; hybridization; and introgression of new alleles from crops or non-indigenous species.

One of the key goals of I*O*MAP is the establishment of diversity panels of wild *Oryza* species for evolutionary studies and conservation efforts. Following in the footsteps of I*O*MAP, ‘the Americas project’ was funded to investigate the present and historic genetic diversity found in wild relatives of rice endemic to North, Central, and South America. There are four wild *Oryza* species endemic to the Americas: one AA genome type—*O. glumipatula* Steud, and three CCDD types—*O. alta* Swallen, *O. grandiglumis* (Doell) Prod, and *O. latifolia* Desv (Vaughan, 1989). Insights into the distribution, diversification, and genetic diversity will inform: (i) the identification of candidate accessions as suitable targets for the neodomestication and/or the genetic improvement of rice crops to respond to environmental challenges due to climate change; and (ii) the establishment of a valuable resource to be used to promote conservation of the wild *Oryza* endemic to the Americas. To accomplish these objectives, we will generate a collection of both contemporary specimens collected in the Americas (with established collaborations with the Universidad de Costa Rica, the Universidade Federal de Pelotas in Brazil, the Universidad de las Americas in Ecuador, and CIAT in Colombia), and herbarium specimens from the Royal Botanical Gardens, Kew (RBG Kew, London, UK), the Natural History Museum (NHM) in London, the New York Botanical Garden (NYBG, New York, USA), the Smithsonian National Museum of Natural History (Washington DC, USA), and the Muséum National d’Histoire Naturelle (MNHN) in Paris. Whole-genome sequencing of historical and contemporary collections, and analysis of single nucleotide polymorphisms (SNPs), will be the starting point to study evolutionary processes to estimate loss or gain of genetic diversity over time, detect hybridization and admixture events between populations/species, and identify candidate regions involved in adaptation to local conditions (Fig. 1). To generate the SNP panel, we will take advantage of the assembled and annotated Platinum Standard Reference Sequences (PSRefSeqs) of the four species previously generated in the Wing lab and deposited in NIH GenBank (Benson *et al.*, 2013; https://www.ncbi.nlm.nih.gov/bioproject/?term=PRJNA48429). The genetic information gained will be useful to evaluate the condition, threat of genetic erosion, and conservation status of wild populations to inform on their long-term viability status and, possibly, the need for conservation/protection strategies. Detection of adaptive variation can be harnessed for future crop improvement and neodomestication efforts (Godfray *et al.*, 2010).

## The wild *Oryza* species in the Americas

*Oryza glumipatula* is the only diploid wild rice species in the New World, and ranges from Central to South America and the Caribbean. It grows in flooded areas, swamps, rivers, and humid areas with clay soils. The life cycle depends on the geographical location of the populations, ranging from perennial to annual or biannual (Karasawa *et al.*, 2007). The plant is characterized by a bushy growth, with fragile stems near the base that can detach and fluctuate on rising water, forming new populations downstream (Vaughan *et al.*, 2003). Flowering occurs predominantly through self-pollination, although cross-pollination is also possible (Fuchs *et al.* 2016; Table 1). There are no clear morphological differences between *O. glumipatula* and AA genomes from Asia and Australia. The Plants of the World Online (POWO), an international collaborative program facilitated by the RBG Kew, considers *O. glumipatula* as a synonym for *O. rufipogon* (https://powo.science.kew.org/results?f=&q=*Oryza*%20glumipatula). However, some sterility barriers occur between *O. glumipatula* and Asian AA species (Juliano *et al.*, 1998), suggesting speciation. Wambugu *et al.* (2015) used whole chloroplast sequences to clarify the phylogenetic relationships among the AA genomes. The phylogenetic tree defined two primary clades: a basal South American/African clade containing *O. glumipatula* and *O. longistaminata*, and a second clade containing all other *Oryza* AA genome species (Wambugu *et al.*, 2015). A phylogenetic analysis of the 13 *Oryza* genome dataset by Stein *et al.* (2018) showed that *O. glumipatula* is more closely related to the African *O. barthii* than to the Asian *O. rufipogon*. Accordingly, clear signatures of introgressions were found in 10 out of 12 chromosomes between *O. glumipatula* and the African species, but not the Asian species (Stein *et al.*, 2018). Reciprocal introgression lines between *O. glumipatula* and *O. sativa* have been developed, and facilitated the identification of genes such as *Rhw*, controlling hybrid weakness restoration, and *S22*, regulating pollen semi-sterility and hybrid sterility (Vaughan *et al.*, 2003). Genetic diversity studies for this species have been carried out in Brazil and Costa Rica: in Brazil, two main groups were found in the Amazon basin and one in the Pantanal (Karasawa *et al.*, 2007); for Costa Rica, two main groups were found—one in the north-east and one in the north-west side of the country (Fuchs *et al.*, 2016). Being predominantly self-pollinated implies a tendency for *O. glumipatula* to lose genetic diversity and increase genetic differentiation among populations, with the risk of fragmentation and lower flexibility in response to environmental changes (Karasawa *et al.*, 2007).

**Table 1.** Brief description of the wild *Oryza* species endemic to the Americas

Species	Karyotype	Genome type	Genome Size (Mbp)	Morhology	Geographical Distribution	Natural Habitat	Traits of interest
***Oryza glumipatula***	24	AA	464	Bushy growth with fragile stem near the base that can detach and float on rising water	Brazil, Colombia, Costa Rica, French Guiana, Cuba, Suriname, and Venezuela	Flooded areas that become seasonally dry, swamps, rivers, and humid areas with clay soils; open and sunny areas	Resistance to blast, *M. graminicola*, and rice black streaked dwarf virus (RBSDV); source of cytoplasmic male sterility (CMS); submergence tolerance
***Oryza*** ***alta***	48	CCDD	895	Tall (up to 4 m), erect plant with broad leaves (generally >5 cm), spikelet length >7 mm	Belize, Brazil, Colombia, Guyana, Paraguay, and Venezuela	Found mainly in savannah, sometimes in woodland, in seasonally flooded habitats; open and sunny areas	Resistance to striped stem borer (SSB); salinity tolerance; high biomass production
***Oryza grandiglumis***	48	CCDD	865	Tall (up to 4 m), broad leaves (3–5 cm), pubescent ligule, sterile lemma	Argentina, Bolivia, Brazil, Colombia, Costa Rica, Ecuador, French Guiana, Paraguay, Peru, and Venezuela	Found in savannah or woodland, in seasonally flooded areas; open and shaded areas	Submergence tolerance; high biomass production
***Oryza latifolia***	48	CCDD	1043	Both short (usually <1 m) and tall (≥2 m) forms exist. Broad leaves, typically <5 cm; spikelet length <7 mm	Argentina, Belize, Bolivia, Brazil, Colombia, Costa Rica, Cuba, Dominican Republic, Ecuador, El Salvador, French Guiana, Guatemala, Guyana, Haiti, Honduras, Mexico, Nicaragua, Panama, Paraguay, Peru, Puerto Rico, Surinam, Trinidad, and Venezuela	Found in low forest, rainforest, open woodland, undulating savannah, pasture, cultivated fields, and open swamp. Often grows in or near water. In seasonally dry, open or semi-open, sunny areas	Resistance to bacterial blight (BB), brown planthopper (BPH), and white-backed planthopper (WBPH); salinity tolerance; high biomass production

Data are from Vaughan (1994), Zamora *et al.* (2003), Bailey-Serres *et al.* (2010), Zhang *et al.* (2015b), Brar and Khush (2018), Prusty *et al.* (2018), and Ray *et al.* (2018).

*Oryza alta*, *O. grandiglumis*, and *O. latifolia* are widely distributed in Central America, South America, and the Caribbean, and show similar morphological characteristics and distribution, with few distinctions. *Oryza alta* (considered a synonym for *O. latifolia* by POWO, https://powo.science.kew.org/results?f=&q=*oryza*%20alta) is a perennial type characterized by tall forms growing up to 400 cm in height, with broad leaves and a long spikelet length. It is found along river basins, streams, lake edges, and canals in deep water, usually in open and sunny areas*. Oryza grandiglumis* is a perennial tall plant with open panicles and a sterile lemma, and grows in savannas as well as woodlands with clay and alluvial soils. Its characteristic culm elongation, which may reach up to 760 cm in height, makes this species suitable to grow in areas subject to seasonal flooding. *Oryza latifolia* is a perennial plant found in aquatic ecosystems (such as swamps, savannas, grassland, and woodlands, and along hill slopes and the shores of rivers and coasts) and its growth depends on seasonal variations and water levels. *Oryza latifolia* shows both dwarf and tall forms, broad leaves, long panicles, high biomass, and a large number of spikelets (Table 1).

The origin of subgenome composition in the CCDD species is controversial. The three CCDD genomes are assumed to track back to a single polyploidization event (Vaughan *et al.*, 2003), where the CC genome (*O. officinalis* or *O. rhizomatis*; Bao and Ge, 2004; Ammiraju *et al.*, 2010b) contributed as the maternal donor. However, the origin of the DD subgenome remains unclear, and the progenitor genome is presumed to be extinct, or yet to be discovered (Vaughan *et al.*, 2003; Shenton *et al.*, 2020). *Oryza australiensis* (genome type EE) has been proposed as the potential donor of the DD subgenome in many studies (e.g. Bao and Ge, 2004; Hirsch *et al.*, 2009). However, the study of Li *et al.* (2001) supported the original cytological definition of a distinct DD genome. Using multicolor genomic *in situ* hybridization (McGISH) in *O. alta* with probes from both the CC (*O. eichingeri* and *O. officinalis*) and EE genomes (*O. australiensis*), the authors found that the EE genome is closer to the CC than to the DD subgenome, and therefore rejected the hypothesis of the EE genome as the direct donor of the DD subgenome (Li *et al.*, 2001).

A succession of phylogenetic studies of the *Oryza* species at different times [e.g. using restriction fragment length polymorphisms (RFLPs), amplified fragmernt length polymorphisms (AFLPs), chloroplast fragments, nuclear genes, and whole-genome SNPs] showed a very close proximity among the three CCDD genomes compared with the other species of the genus, with *O. alta* and O*. grandiglumis* more closely related to each other than to *O. latifolia* (Wang *et al.*, 1992; Aggarwal *et al.*, 1999; Bao and Ge, 2004; Shenton *et al.*, 2020).

Although highly related, the CCDD genome species appear to have diversified in relation to different ecological conditions. For example, field reports from Shimamoto, Ohara, and Akimoto in the Rio Solimões (in the Amazon state) describe an atypical population of *O. grandiglumis* showing some morphological characteristics of *O. alta*. Oliveira *et al.* (1994) reported a population in the region of Iranduba (Amazon state) comprising both typical specimens of *O. grandiglumis* and *O. alta*, and specimens displaying intermediate forms. In the region of Santarem (Parà state), they found a population of *O. alta* showing morphological deviations towards *O. grandiglumis* (Oliveira *et al.*, 1994). Such field observations highlight the importance of collecting genetic and ecological information on these species over a range of ecotypes to help in locating the number and size of populations for appropriate conservation strategies (Vaughan *et al.*, 2003). This topic will be discussed further below.

## Wide range of resistances to stress

Genomic and genetic research of rice cultivars and their wild relatives has greatly advanced the understanding of resistance traits for crop improvement. The wild *Oryza* species in the Americas possess many naturally occurring resistance/tolerance traits, such as resistance to biotic diseases caused by bacteria [e.g. bacterial blight (BB)], insects [e.g. brown planthopper (BPH)], fungi (e.g. blast), and nematodes (e.g. *Meloidogyne graminicola*), and tolerance to abiotic stresses (e.g. flooding and salinity), as shown in Table 1.

BB, caused by the bacterium *Xanthomonas oryzae* pv. *oryzae* (*Xoo*), is widespread in almost all rice-growing countries and can cause yield loss of up to 50% depending on the variety, growth stage, geographic location, and environmental conditions of the rice crop (Jiang *et al.*, 2020). According to Jiang *et al.* (2020), >40 resistance genes conferring host resistance to various strains of *Xoo* have been identified in rice [e.g. receptor-like kinase (RLK) genes, sugar will eventually be exported transporter (SWEET) genes, and executor genes]. The RLK gene *Xa21*, which originated from *O. longistaminata*, was the first cloned resistance gene in rice (Song *et al.*, 1995). Subsequently, interspecific crosses between an elite breeding line of *O. sativa* and *O. latifolia* resulted in new lines showing resistance to BPH and BB (Multani *et al.*, 2003).

BPH (*Nilaparvata lugens* Stål) has become a severe constraint in rice production. Between 2005 and 2008, it caused a combined production loss of 2.7 Mt of rice in China (Brar *et al.*, 2009). This migratory herbivore is a vector for plant viruses. An outbreak of BPH in Vietnam in 2005 caused the transmission of ragged stunt virus (RRSV) and rice grassy stunt virus (RGSV), resulting in the loss of 828 000 t of rice (Cabauatan *et al.*, 2009). The most adopted method to control BPH is the application of pesticides, whose intensive use has led to environmental pollution, the death of pests’ natural enemies, and the development of insecticide-resistant BPH populations. Host plant resistance is therefore a more desirable, sustainable, and economic strategy for the control or management of BPH (Hu *et al.*, 2016). Qiu *et al.* (2012) constructed pyramid lines containing the partially dominant BPH12 gene from *O. latifolia*, and the BPH6 gene. The combination of the two BPH resistance genes resulted in an additive effect and overall increase in resistance compared with single-gene-resistant lines (Qiu *et al.*, 2012).

*Magnaporthe oryzae*, the aetiological agent of blast, is a devastating fungal disease that infects rice plants from the vegetative (leaf blast) to the reproductive stage (neck blast), and can cause up to 30% of crop loss annually (Devi *et al.*, 2020). This pathogen evolves rapidly and presents species- and cultivar-specific races. According to Li *et al.* (2019), ~100 resistance genes (Pi genes) and 500 quantitative trait loci (QTLs) associated with blast resistance have been identified in rice. Due to the high variability of the fungus and the fast breaking down of resistant lines, breeders prefer varieties that contain multiple resistance genes and have broad-spectrum blast resistance (Li *et al.*, 2019). Devi *et al.* (2020) characterized a major effect QTL from *O. glumipatula*, qBL3, and identified a putative candidate gene, Pi68(t), conferring field resistance to leaf and neck blast (Devi *et al.*, 2020).

Root-knot nematodes (RKNs) are damaging parasites of the genus *Meloidogyne* infecting roots of many plants, including rice. RKN *M. graminicola* is widespread in subtropical and tropical regions, and is responsible for yield losses ranging from 11% to 80% in irrigated rice systems in Asia and the Americas (Mattos *et al.*, 2019). Given that resistance to *M. graminicola* reported in *O. sativa* is limited, Mattos and colleagues evaluated resistance and response mechanisms to this RKN in the American wild rice species. *Oryza glumipatula* showed high levels of resistance to infection of *M. graminicola*, suggesting it as a potential source of resistance to this nematode in breeding programs, whereas *O. alta* and *O. grandiglumis* were found to be moderately resistant and susceptible, respectively (Mattos *et al.*, 2019).

Both *O. glumipatula* and *O. grandiglumis* show submergence tolerance traits (Hattori *et al.*, 2009; Okishio *et al.*, 2014; Dos Santos *et al.*, 2017). There are two known coping mechanisms to flooding in rice: an escape response to prolonged submergence at the mature stage, where internode elongation allows the plant to progressively emerge and float on the water; and a quiescent response, where the shoot growth is reduced during flash floods in completely submerged seedlings and resumes to normal growth after the flood recedes (Gumi and Aliero, 2018). Floating ability during the escape response in rice is driven by two ethylene response factors (ERFs), namely SNORKEL1 (SK1) and SNORKEL2 (SK2), whose expression induces rapid internodal elongation in response to ethylene in *O. sativa* (Hattori *et al.*, 2009). Although SK genes were not found in *O. grandiglumis*, this species showed enhanced elongation of internodes upon rising water levels, suggesting an SK-independent internode elongation mechanism to prolonged flooding (Okishio *et al.*, 2014). *Oryza glumipatula* responds to deep water conditions and was shown to possess SK2 and SK2-like genes, but not SK1 (Hattori *et al.*, 2009), indicating the importance of SK2 in prolonged submergence response. A different accession of *O. glumipatula* possessing both SK1 and SK2 homolog sequences was used by Sasayama *et al.* to investigate submergence response induced by hypoxia. The authors found that hypoxia-induced internodal elongation in this accession occurred even when ethylene biosynthesis was inhibited, and suggested hypoxia as the first signal of elongation growth in this species (Sasayama *et al.*, 2018). The submergence tolerance through a quiescent response is associated with the SUB1 locus (Xu and Mackill, 1996), which is conserved on chromosome 9 in many *Oryza* species including the AA, BB, and FF genome types (Dos Santos *et al.*, 2017). Xu *et al.* (2006) sequenced this region in the tolerant cultivar FR13A and found three ERF-encoding genes, namely SUB1A, SUB1B, and SUB1C. Overexpression of the allele SUB1A-1 in flooding-susceptible *O. sativa* varieties showed that this allele is sufficient to induce a flooding survival response. Accessions carrying the SUB1A-2 allele are sensitive to this stress (Xu *et al.*, 2006). Although *O. grandiglumis* survives complete submergence, Okishio and colleagues could not find homologs of the SUB1A gene in *O. grandiglumis* (Okishio *et al.*, 2014), and found that submergence tolerance response in this species is not regulated by ethylene (Okishio *et al.*, 2015). Interestingly, SUB1B- and SUB1C-like genes were found in *O. glumipatula*, whereas a SUB1A-like gene sequence was not detected (Dos Santos *et al.*, 2017).

Prusty *et al.* (2018) evaluated salt stress tolerance in wild *Oryza* species, comparing them with cultivated salt-tolerant and sensitive lines. The evaluation was based on many factors, including a visual salt injury score, number of days of seedling survival, shoot length and biomass, chlorophyll content, leaf and root ion content, and level of expression of salt tolerance genes (i.e. *OsHKT1;4*, *OsHKT1;5*, and *OsSOS1*) under stress conditions. All CCDD genomes exhibited salt stress tolerance properties, with *O. alta* and *O. latifolia* showing similar levels of tolerance to *O. coarctata* (a well-known halophyte in the genus), followed by *O. grandiglumis* and other wild species (Prusty *et al.*, 2018).

## Biodiversity conservation

One of the main foci of I*O*MAP is the identification of populations of wild *Oryza* species for diversity analysis and conservation efforts (Jacquemin *et al.*, 2013). Rice crop improvement via crop wild relatives (CWRs) is contingent on the effective availability and conservation of these genetic resources (Thomas *et al.*, 2017). Conservation of1 diversity for CWRs can be achieved through: (i) *ex situ* conservation strategies (i.e. conservation in seed banks, herbaria collections, or artificial environments reproducing conditions of the original environment) and (ii) *in situ* conservation strategies (i.e. conservation of CWRs within their original species range) (Vincent *et al.*, 2019).

**Fig. 2.**
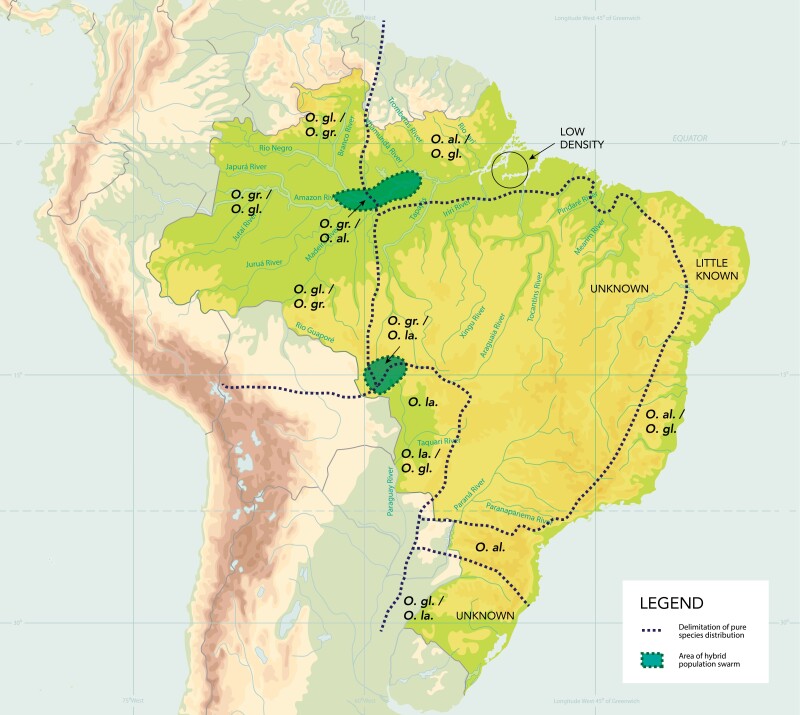
The map depicts the geographical distribution of the wild *Oryza* species in Brazil as described by Giancarlo C.X. Oliveira after his field trips to the Amazon in 1988, 1992, and 1993 and a survey of Brazilian herbaria. *O. gl*., *O. glumipatula*; *O. gr*., *O. grandiglumis*; *O. al*, *O. alta*; *O. la*, *O. latifolia*. Limits of species distribution are based on Oliveira GCX, Morishima H, Martins PS. 1994. Investigations of plant genetic resources in the Amazon basin with the emphasis on the genus *Oryza*: Report of 1992/93 Amazon Project—funded by FAPESP (São Paulo State Foundation for the Support of Research) and the Ministry of Education of Japan, with permission.

Germplasm collections of wild and cultivated rice species started in the 1950s. During the Green Revolution in India and the Philippines in the late 1960s, crop improvement programs boosted the collections of landrace rice germplasm by the international scientific community (Vaughan *et al.*, 2003). Since the 1960s, the International Rice Research Institute (IRRI) in the Philippines has carried out programs for the genetic improvement of rice, has maintained an international collection of rice genetic resources (IRGC) for germplasm preservation, and has provided seeds freely for scientific research purposes (Evenson and Douglas, 1997). The Germplasm Resource Information Network (GRIN) database indicates that IRRI currently maintains seed resources for 13 accessions of *O. alta*, 52 of *O. glumipatula*, 9 of *O. grandiglumis*, and 77 of *O. latifolia* (https://gringlobal.irri.org/gringlobal/search). A program for botanical exploration and collection of wild crop relatives, rice included, was initiated in Brazil after the establishment of the Brazilian Agricultural Research Corporation (Embrapa) in 1973. CWRs of rice have been conserved in the Embrapa’s Rice Genebank since 1992, with 177 accessions of *O. alta*, *O. glumipatula*, *O. grandiglumis*, and *O. latifolia* present in 2015 (Medeiros *et al.*, 2021). In 2002, Morishima collected the reports made by the Japanese National Institute of Genetics between 1957 and 1997, describing field trips and observations of wild and cultivated rice species around the world, including the Americas (Fig. 2). In the preface of his book, he claims that habitats and populations of rice species described therein changed or were lost since the time of the initial expeditions (Morishima, 2002).

The characterization of ecological and genetic aspects of a species is necessary to efficiently prioritize populations for conservation. An extended definition of conservation biology includes the application of genomics to conservation (Allendorf *et al.*, 2010). Through increasingly accessible genomics technologies, the genotyping of hundreds of thousands of loci in large sample sizes improves the estimation of the parameters widely used in conservation genetics, such as population diversity, structure, effective size, and demographic history (i.e. the variation of population size through time). This information is crucial to define units of conservation for diversity protection and management plans (Allendorf *et al.*, 2010).

Medeiros *et al.* (2021) described the conservation status of four native species related to crops in Brazil, which included rice. They used 130 germplasm accessions of the four wild *Oryza* species conserved *ex situ* and 112 herbarium records to evaluate the status of *ex situ* conservation, and collected 47 new accessions in five sites in the Pantanal and the Amazon to evaluate the status of *in situ* conservation. This study was carried out within a global initiative supported by the Government of Norway, and managed by the Global Crop Diversity Trust and the Millennium Seed Bank at the RBG Kew, whose goal is the establishment of national programs for the collection and *ex situ* preservation of prioritized germplasm. The authors estimated gaps of representativeness of the four *Oryza* species in *ex situ* collections, and highlighted the need to develop complementary *in situ* conservation strategies: one proposed action is the expansion and consolidation of protected areas in the Cerrado region, which currently show a narrow geographical range and are under threat of high anthropogenic impact (Vincent *et al.*, 2019; Medeiros *et al.*, 2021).

Anthropogenic activities such as conversion of land to agriculture, and, in general, human intervention in natural patterns and processes are associated with threats to population viability and biodiversity conservation (e.g. changes in the environment, disturbance of the interaction with other species, and genetic threats). These threats can lead to different outcomes, such as habitat fragmentation, or gene flow from non-indigenous or crop species (Fig. 1). Habitat fragmentation implies the shattering of a continuous habitat with a progressive isolation of populations, and generally leads to a decrease in population size and decrease in migrations among populations. This results in reduced genetic diversity and lower adaptive capability and overall fitness. Between-species hybridization can lead to introgression and reduced genetic integrity of the native species, especially in small populations which tend to behave as sinks (Oostermeijer, 2003). Using AFLPs, Fuchs *et al.* found indirect evidence that *O. glumipatula* individuals were admixed with cultivated *O. sativa* varieties. The risk of losing local genetic diversity via introgression in the largest population of *O. glumipatula* in Costa Rica led the authors to highlight the importance of developing *in situ* and *ex situ* conservation strategies for this rice wild relative (Fuchs *et al.*, 2016). The effect of climate change on ecosystems can alter the geographic connectivity of species and populations, and affect the amount of gene flow among them (Huang and Schaal, 2012). Thomas *et al.* (2017) assessed the genetic diversity, population structure, and natural distribution range of the wild relatives of rice in Colombia. They estimated the spatiotemporal overlap between geographic areas suitable for the growth of wild and cultivated rice under current and predicted future climate conditions, and assessed the potential scale of gene flow from the latter to the former. They found that all the wild relatives, except *O. latifolia*, are predicted to experience range expansion under climate change. On the other hand, most habitats suitable for the four wild rice species are located outside protected areas in Colombia, hampering an effective *in situ* conservation program in areas with high risk of alien introgression from cultivated rice. The authors suggested that effective strategies for *ex situ* and *in situ* conservation in Colombia should consider the prioritization of populations showing the highest level of genetic diversity, complementarity in terms of genetic differentiation (i.e. population units), and adaptive traits (Thomas *et al.*, 2017).

## The neodomestication of *Oryza alta*

Neodomestication has revolutionized the concept of crop improvement by shifting the focus of incremental gains in domesticated species to giant leaps forward in CWRs. The core of the technique resides in the editing of domestication-related genes in a CWR to obtain novel genotypes with the desired agronomic characteristics while keeping the natural genetic diversity and adaptive traits of the CWR (Zhu and Zhu, 2021). Recently, a successful neodomestication of a wild rice tetraploid was accomplished in *O. alta* (Yu *et al.*, 2021). Yu and colleagues identified gene homologs of domestication-related genes in *O. alta* that regulate important domestication (e.g. seed shattering, awn length, hull and pericarp color, panicle shape, and grain width), and agronomic traits in rice cultivars (e.g. genes regulating yield, grain quality, efficient use of nutrients, and resistance to biotic and abiotic stresses) as targets for gene editing. The essential tools required to accomplish this task were: (i) a high-quality annotated reference genome to enable the identification of target genes, and (ii) a genotype that is suitable for regeneration and genome editing. Yu *et al.* developed a protocol for tissue culture and genetic transformation of *O. alta*, and implemented a genome editing system that uses CRISPR/Cas9. Subsequently, Yu *et al.* improved yield in *O. alta* by inducing a mutation in an ortholog of *O. sativa GS3*, a gene that regulates grain length in diploid rice. Spontaneous seed shattering was inhibited by targeting a QTL associated with abscission layer formation (i.e. qSH1), an essential and ancient trait found in almost all domesticated cereals. The Green Revolution gene *SD1*, which controls plant height with shorter culms and consequential resistance to lodging, was also edited. Combining improved domestication- and agricultural-related traits with natural adaptation to native environments can be obtained at an unprecedented speed compared with traditional techniques involving breeding and introgression. Through their work, Yu and colleagues showed a route for the generation of a new crop in a wild tetraploid *Oryza* species that opens the door to the exploitation of other wild polyploid genomes to support global food security.

## Conclusions

The generation of a vast array of publicly available genomic resources by (I)*O*MAP starting two decades ago helped the rice scientific community to address complex biological questions on a whole-genome scale, such as the dynamics of the evolution of the genus, the impacts of domestication, the diversity and evolution of gene families useful for crop improvement (Wing *et al.*, 2007; Jacquemin *et al.*, 2013), etc. The main objective of ‘I*O*MAP: the Americas’ is thus to provide a valuable genomic asset describing the genetic diversity, population structure and demographic history, gene flow, and potential hybridization of populations of wild rice species endemic to the Americas. Putative candidate regions for adaptive traits, such as resistance to pathogens and tolerance to abiotic stress, will be identified and explored. The genomic characterization of these species will help the identification of accessions as potential candidates for future neodomestication efforts and the genetic improvement of stress-sensitive rice crops. The effect of climate change is predicted to modify the distribution ranges of the wild relatives of rice in the Americas (Thomas *et al.*, 2017), and to alter inundation patterns in western Amazonian rivers with significant consequences for lowland agriculture, including cultivation of rice (Coomes *et al.*, 2016). The adaptive capacity of the wild *Oryza* species in the Americas may be exploited to cope with effects of climate change on the environment. For example, neodomesticated submergence-resistant *O. glumipatula* and *O. grandiglumis* could be cultivated in their native aquatic and flooded habitats in the Amazon basin to replace non-adapted rice cultivars; neodomesticated salt-tolerant *O. latifolia* and *O. alta* could be cultivated in areas where the rising sea level and reduced freshwater flow from upper catchments is causing severe saltwater intrusion, soil salinity increase, and damage to rice crop production, similar to that reported for the Mekong Delta River in Vietnam and the Satkhira River in Bangladesh (Radanielson *et al.*, 2018; Paik *et al.*, 2020). The generation of a vast diversity panel spanning a large geographic area and a temporal range of decades will inform the potential erosion of genetic variation in the populations/species due to climate change, human activity, and hybridization events, focusing conservation efforts where they are needed most, and facilitating future efforts of comparative evolutionary genomics studies. Eventually, we trust that the knowledge of the genetic diversity and health status of the wild *Oryza* species endemic to the Americas will help identify the most appropriate accessions to include in *ex situ* core collections and the development of guidelines for conservation of biodiversity *in situ* that are sustainable. Rather than establishing protected reserves that preserve species’ presence only, future research on the wild relatives of rice should be applied to establish reserves based on genetic diversity, as proposed by Vincent *et al.* (2019), with suitable management plans aimed at the maximization and maintenance (or even enhancement) of the genetic diversity of priority rice specimens.

## Abbreviations

BB: bacterial blight
BPH: brown planthopper
CWR: crop wild relative
I*O*MAP: International *Oryza* Map Alignment Project
IRGSP: International Rice Genome Sequencing Project
LTR-RT: long terminal repeat retrotransposable element
MY: million years.
